# Supplementary material for: Persistent epigenetic memory impedes rescue of the telomeric phenotype in human ICF iPSCs following DNMT3B correction
Source: eLife. 2019 Nov 20;8:e47859. doi: 10.7554/eLife.47859 (PMC6897513; doi:10.7554/eLife.47859)
Supplement: Supplementary file 1. [file elife-47859-supp1.docx]

**Supplementary Table 1**

**Primers for bisulfite analysis by Ion torrent PGM**

| **Reverse primer^a^** | **Forward primer^a^** | **Chromosomal positions in Human GRCh38** | **Region** |
| --- | --- | --- | --- |
| TAAAATTCCACCTATCTCTATAC  TGAGGTTCCACCTGTCTCTGTGC | GTATTGTAGGTGTATAGTTGTATAAG  GTACTGCAGGTGCACAGCTGCATAAG | **Chr2**:11,333-11,460 | **Subtelomere 2p** |
| AAAAACCAAATAAAACAACAAA  AGAGGCCAAATAGAACAACAAG | GAGGGTGGAATTTTAGTAATTT  GAGGGTGGAACCTCAGCAATCT | **Chr5**:13,176-13,366 | **Subtelomere 5p** |
| CCATAAATAATCAAAATAAAAAACC  CCATGAATAATCAAGGTGAGAGACC | TTATGTGTGTATTAGGAATGTTGTA  TCATGTGTGCATTAGGAATGCTGCA | **ChrX**:156,029,669-156,029,860  **Chr9**:10,507-10,681 | **Subtelomeres 9p/Xq** |
| CTAACTTTTCAAATTACTAAAATTC  CTGGCTTTTCAGATTACTGAGGTTC | TGGTGTAGATGTAGAGAAGA  TGGCGCAGACGCAGAGAAGA | **Chr4**:11,545-11,748 | **Subtelomere 4p^b^** |
| AAAACTATATTCTCATCAACAAAAAC  GAAGCTGTGTTCTCATCAGCAGAGAC | GTAAGGTTTGTGTTGATTAGGA  GCAAGGTCTGTGCTGATCAGGA | **Chr18:** 10,732-10,916  **Chr10:** 10,536-10,720 | **Subtelomeres 10p/18p** |
| TCTACACAACCTTTTAAAATAC  TCTGCACAGCCTTTTGGGGTAC | TTTTTAAGGTTTGTGTTGAGG  CCCCCAAGGTCTGTGCTGAGG | **Chr7**: 159,335,309-159,335,467 | **Subtelomere 7q** |
| AAAAACAAACAATACCCCCAAC  AAAGGCAGGCAGTACCCCCAAC | GAGTAGAGTTTTTTTTAGGTTAGATT  GAGCAGAGTTCTTCTCAGGTCAGACC | **Chr**[**10**:133,786,654-133,786,804](https://www.ensembl.org/Homo_sapiens/Location/View?r=10:133786646-133786811;tl=ZPHqcFp26x5GTtnv-4259339-693267756)  **Chr**[**4**:190,204,097-190,204,247](https://www.ensembl.org/Homo_sapiens/Location/View?r=4:190204089-190204254;tl=ZPHqcFp26x5GTtnv-4259339-693267758)  **Chr**[**2**:242,182,895-242,183,045](https://www.ensembl.org/Homo_sapiens/Location/View?r=2:242182887-242183052;tl=ZPHqcFp26x5GTtnv-4259339-693267757) | **Subtelomeres 2q/4q/10q** |
| AAACACTACAAAACCAACTTACCC  GGGCACTACAGGACCAGCTTGCCC | TGTTGTTATTATAATGTGAGGAAGAGG  TGCTGCCACTATAATGTGAGGAAGAGG | **Chr10**:133,785,955-133,786,173  **Chr2**:242,182,046-242,182,264  **Chr4**:190,203,253-190203,471  **Chr13**:114,352,791-114,353,009  **Chr1**:248,944,437-248,944,655  **Chr21**:46,698,328-46,698,546 | **Subtelomeres 10q, 1q/21q, 2q/4q, and 13q^c^** |
| ACTACG/ATTCTATTCAACACAAAC  GCTGCGTTCTGTTCAGCACAGAC | TAATTGGTTTTTGATTTTGATT  TAACTGGTCTCTGACCTTGATT | **Chr10:**133,786,714-133,786,885  **Chr2:**242,182,955-242,183,126  **Chr4:**190,204,157-190,204,328 | **Subtelomeres 10q, 2q/4q^d^** |
| TAAATAATTCCATTCG/AAATCC  TAGATGATTCCATTCGGGTCC | TGGAATTATTTTTTAATGGAAA  TGGAATCATCTTCTAATGGAAA | **Chr1**: 125,180,136- 125,180,294  **Chr1**: 143,215,669- 143,215,827  **Chr1**: 143,192,699- 143,192,857 | **Satellite 2** |

**^a^**Bisulfite converted primers are in black. Sequence in gray depicts the genomic regions prior to bisulfite conversion

**^b^** These primers amplify an additional region in 4q- Chr4:190,120,841-190,121,044 positioned at least 30kb from chromosome end (based on UCSC)

**^c^** These different subtelomeres were told apart based on the following single nucleotide differences compared to subtelomere 10q (nucleotide number in amplicon):

| Subtelomere | Nucleotide #1 | Nucleotide #2 |
| --- | --- | --- |
| 1q/21q | 121C>G | 137G>A |
| 2q/4q | 128C>G | - |
| 13q | 162G>C | 179C>A |

**^d^** These primers were used following treatment with OICR9429. The different subtelomeres were told apart based on the following single nucleotide differences compared to subtelomere 10q (nucleotide number in amplicon):

| Subtelomere | Nucleotide #1 | Nucleotide #2 |
| --- | --- | --- |
| 2q/4q | 39G>A | - |
