# Supplementary material for: Persistent epigenetic memory impedes rescue of the telomeric phenotype in human ICF iPSCs following DNMT3B correction
Source: eLife. 2019 Nov 20;8:e47859. doi: 10.7554/eLife.47859 (PMC6897513; doi:10.7554/eLife.47859)
Supplement: Supplementary file 2. [file elife-47859-supp2.docx]

**Supplementary Table 2**

**TERRA promoter consensus repeats utilized for alignments with WGBS data**

| **61 bp repeats sequences** | |
| --- | --- |
| 61-1 | GCCACTACTGTTGGAGCAAGAGGGCCCAGCAGTGTCCCCAGCTGCCAGCAGGCGGGCGTGCT |
| 61-2 | GCCAGTACACCTTGAGCAAGAGGACCCTGCAATGTCCGTAGCTGCCAGCAGGCGGCGT |
| 61-3 | GCCACCACTATACAGTAAGCAAGAGGACCCTGCAGTGCCCCGGCGCCACGAGGGGGCGGT |
| 61-4 | GGCCACCACTCTAAGCAAGAGAGCCCTGCAGTTGCCCTAGTCGCCAGCAGGGGGCGCCCT |
| 61-5 | GGCACAGCACCGTGAGCAAGCGGGTCCTGTAGTGCCCGGCTGCAAGCAAGGGGCGGTCGAT |
| 61-6 | GCCGTTACGCCGGGAGCAAGAGGGCCCTGCGTAGTCCCCATCTGCCTGCATGTGGCGTGCA |
| 61-7 | GCCACGACAATGGCAGCAAGAGGGCCCGGCAGTGTGCCCAGCTGCCAGCAGGCGGGTGTGCT |
| 61-8 | GCCACTATAATGTGAGGAAGAGGGCCCTGCAATGTCCCTAGCTGCCAGCAGGCGGCGT |
| 61-9 | GCCACCACTATACTGCGAGCAAGAGAGCCCTGCCGTGCCCCGGCGCCAGCAGGGGGCGCT |
| 61-10 | GGACAGCACTGTAAGCAAGAGGGCCCTGCAGTTGTCCTAGTCGCCAGTAGGGGACGCAAT |
| 61-11 | GGCAGAGCACCGTGGGCAAGCTGGTCCTGTAGTGCCCGGCTGCAAGCAGGGGGCGC |
| **29 bp repeats sequences** | |
| 29-1 | TCTCTGCGCCTGCGCCGGCGCGGCGCGCC |
| 29-2 | TCTCTGCGCCTGCGCCGGCGCGCCGCGCC |
| 29-3 | TCTCTGCGCCTGCGCCGGCGCCCCGCGCC |
| 29-4 | TCTCTGCGCCTGCGCCGGCGCGCCGCC |
| **37 bp repeats sequences** | |
| 37-1 | GCGAGGGCGGAGTTGCGTTCTCTTTAGCACACAC |
| 37-2 | ATCGCGAGGGCGGAGCTGCGTTCTCCTCTGCACAGAC |
| 37-3 | ATTGCGAAGGCGGAGCAGAGTTCTTCTCAGGTCAGAC |
| 37-4 | ACTGCGAGGGTGGAGCTGCGTTCTGTTCAGCACAGAC |
| 37-5 | ACCGTAAAGGCGGAGCAGCATTCTTCTCAGCACAGAC |
| 37-6 | GCGACGGCCGAGTTGCGTTCTCGTCAGCACAGAG |
| 37-7 | ACCGCGAGGGCGGAGCTGCGTTGTCCTCTGCACAGAT |
| 37-8 | ACTGCGAAGGCGGAGCAGAGTTCTCCTCAGGTCAGAC |
| 37-9 | ACCGCGAGGGCGGAGCTGCGTTCTGCTCAGTACAGAC |
| 37-10 | ACCGTAAAGGTGGAGCAGCATTCCCCTAAGCACAGAC |

**^a^**Based on TelBam and TelSau repeats (Brown et al. 1990)
