# Supplementary material for: Persistent epigenetic memory impedes rescue of the telomeric phenotype in human ICF iPSCs following DNMT3B correction
Source: eLife. 2019 Nov 20;8:e47859. doi: 10.7554/eLife.47859 (PMC6897513; doi:10.7554/eLife.47859)
Supplement: Supplementary file 3. [file elife-47859-supp3.docx]

**Supplementary Table 3.**

**Primers for RT-qPCR and ChIP analyses**

| **Referred to in**  **this paper as:** | **Primer sequence** | **Use of these primers** | **Previous references** |
| --- | --- | --- | --- |
| Subtelomere 2p-P | F- GTGGAACCTCAATAATCCGAAAA  R- GGACACCACTGTAAGCAAGATAGC | ChIP | - |
| Subtelomere 2p-D | F- CGCATCGACGGTGAATAAAA  R- GCCTAACTCGTGTCTGACTTTGAG | ChIP and RT-qPCR | (Sagie, Toubiana, et al. 2017) |
| Subtelomere 5p-P | F- TGGCACAGCATCGTAGACAAG  R- TGGAACCTCAGCAATCTGAAAA | ChIP | - |
| Subtelomere 5p-D | F- GAGTGCATTAGCATACAGGT  R- TCCTAATGCACACGTAACAC | ChIP and RT-qPCR | (Sagie, Toubiana, et al. 2017) |
| Subtelomere 10q/13q/19q | F- GGCGCTGGACACCACTGTA  R- TGAGTAATCTGAAAAGCCCGTTT | ChIP | - |
| Subtelomere 2q/10q/13q | F- AACCTGAACCCTAACCCTCC  R- ATTGCAGGGTTCAAGTGCAG | ChIP and RT-qPCR | (Sagie, Toubiana, et al. 2017) |
| Subtelomere 10p/18p | F- CCTTCTAACTGGACTCTGAC  R- GCCACAGCGACGGTAAATAA | ChIP and RT-qPCR | - |
| Subtelomere 1**1**q | F- CCAGCTGCCAGCAGTCGG  R- TTGCTCGCAGTATAGTGG | ChIP | - |
| Subtelomere 11q | F- CTGATTATTCAGGGCTGCAAA  R- GCCGCATCGACGGTGAATAA | RT-qPCR | (Arnoult, Van Beneden, and Decottignies 2012) |
| Subtelomere 7q | F- TTCAGACGGGCTTTTGGTTT  R- ATGGTGAATACAATCCTTTCTGTTTG | ChIP and RT-qPCR | (Sagie, Toubiana, et al. 2017) |
| Subtelomere 9p | F- GGGCGCATTAACGGTGAATA  R- CCGCACTGAACCGCTCTAAC | RT-qPCR | (Sagie, Toubiana, et al. 2017) |
| Subtelomere 15p | F- AACCCTAACCACATGAGCAACG  R- CTCGCCTTAGCTTGGGAG | RT-qPCR | - |
| Subtelomere 19q | F - CTATTGCGAAGGCGGAGC  R – CCCCAACCCCAACCCCAACGGC | ChIP and RT-qPCR | - |
| β-actin | F- TGTACGCCAACACAGTGCTG  R- GCTGGAAGGTGGACAGCGA | Normalizing gene for  RT-qPCR | - |
| Satellite 2 | F- AGTGGAATCATCTNRAATGGA  R- CATTCGAGTCCATTCGATGATT | ChIP | - |
| Hoxa 7 TSS | F- TACTCACCTCCCCTTTCCCA  R- GTACCCTGCTCAGCTCCATC | Negative control region for H3K4me3 ChIP | - |
| GAPDH promoter | F- AAAGCCCGCACCAACCAT  R- AGTCCCTGACCCTGCCTTTC | Negative control region for H3K9me3 ChIP | - |
| Myoglobin exon2 | F- GTGCCAGGGGCTTAATCT  R- GCGTCTGAGGACTTAAAGAAGC | Negative control region for H3K36me3 ChIP | (Gatto et al. 2017) |
